# Supplementary material for: Growth Regulators Improve Outcrossing Rate of Diverse Rice Cytoplasmic Male Sterile Lines through Affecting Floral Traits
Source: Plants (Basel). 2022 May 12;11(10):1291. doi: 10.3390/plants11101291 (PMC9148114; doi:10.3390/plants11101291)
Supplement: Supplementary file 1 [file plants-11-01291-s001.zip › plants-1710629-supplementary.pdf]

CMS Lines	Untreated control (T1)	Combination of GA_3 + IAA + NAA (T3)
**L1 (A1×B1)**	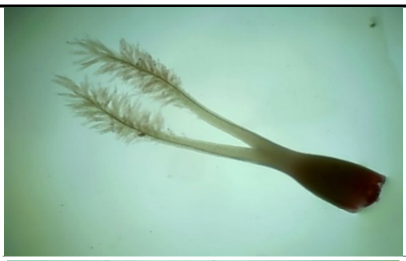	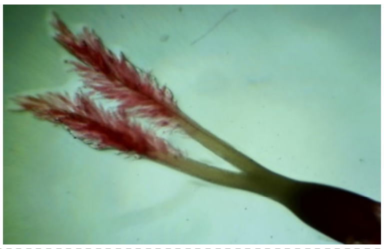
**L2 (A2×B2)**	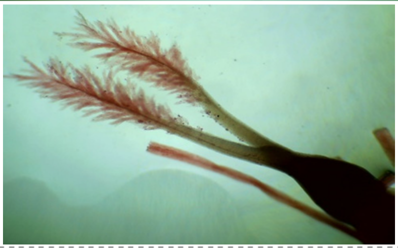	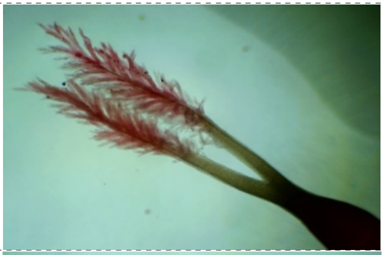
**L3 (A3×B3)**	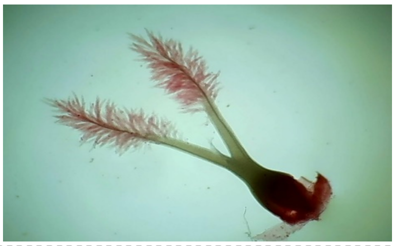	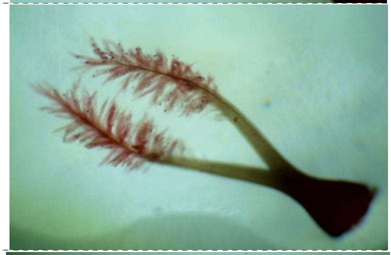
**L4 (A4×B4)**	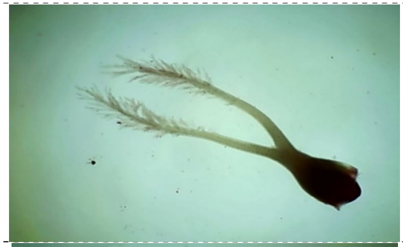	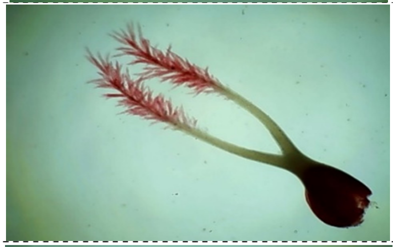
**L5 (A5×B5)**	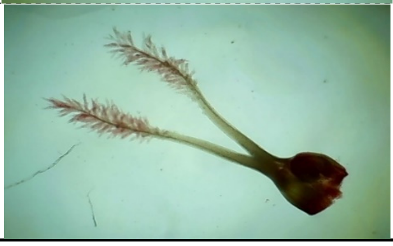	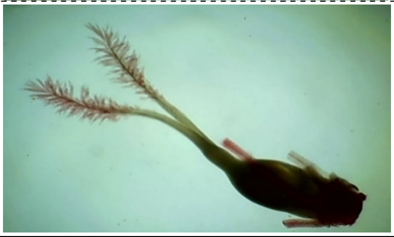

Figure S1. Impact of growth regulators on rice stigma compared to untreated control, scale bar 2 mm.
